# The *ldhA* Gene Encoding Fermentative l-Lactate Dehydrogenase in *Corynebacterium Glutamicum* Is Positively Regulated by the Global Regulator GlxR

**DOI:** 10.3390/microorganisms9030550

**Published:** 2021-03-06

**Authors:** Koichi Toyoda, Masayuki Inui

**Affiliations:** 1 Research Institute of Innovative Technology for the Earth (RITE), 9-2 Kizugawadai, Kizugawa, Kyoto 619-0292, Japan; toyo51@rite.or.jp; 2 Division of Biological Science, Graduate School of Science and Technology, Nara Institute of Science and Technology, 8916-5, Takayama, Ikoma, Nara 630-0192, Japan

**Keywords:** corynebacterium glutamicum, lactate dehydrogenase, GlxR, transcriptional regulation

## Abstract

Bacterial metabolism shifts from aerobic respiration to fermentation at the transition from exponential to stationary growth phases in response to limited oxygen availability. *Corynebacterium glutamicum*, a Gram-positive, facultative aerobic bacterium used for industrial amino acid production, excretes l-lactate, acetate, and succinate as fermentation products. The *ldhA* gene encoding l-lactate dehydrogenase is solely responsible for l-lactate production. Its expression is repressed at the exponential phase and prominently induced at the transition phase. *ldhA* is transcriptionally repressed by the sugar-phosphate-responsive regulator SugR and l-lactate-responsive regulator LldR. Although *ldhA* expression is derepressed even at the exponential phase in the *sugR* and *lldR* double deletion mutant, a further increase in its expression is still observed at the stationary phase, implicating the action of additional transcription regulators. In this study, involvement of the cAMP receptor protein-type global regulator GlxR in the regulation of *ldhA* expression was investigated. The GlxR-binding site found in the *ldhA* promoter was modified to inhibit or enhance binding of GlxR. The *ldhA* promoter activity and expression of *ldhA* were altered in proportion to the binding affinity of GlxR. Similarly, l-lactate production was also affected by the binding site modification. Thus, GlxR was demonstrated to act as a transcriptional activator of *ldhA*.

## 1. Introduction

*Corynebacterium glutamicum* is a nonpathogenic, Gram-positive, facultative anaerobic bacterium harboring a high GC content and belonging to the suborder Corynebacterineae within the class Actinobacteria. It is an important industrial bacterium that is widely used for amino acid production, including glutamate and lysine [1,2]. Complete genomic DNA sequencing, understanding of bacterial metabolism and its regulation, and development of genetic tools for metabolic engineering have expanded the portfolio of bioproducts by *C. glutamicum* and have made this bacterium a promising platform for microbial production of valuable products, including organic acids, biofuel, proteins, commodity chemicals, and healthcare products [3,4,5,6,7,8,9].

The high productivity of several such products is achieved by virtue of the ability of this bacterium to metabolize sugars without growth under oxygen deprivation. On the basis of these properties, we developed a bioprocess in which cells are packed at high cell density to create an oxygen-limiting environment by their own respiration [10]. As proof of concept, we achieved the highest known productivity of organic acids and amino acids via this bioprocess [11,12,13,14].

During the transition from the aerobic exponential growth phase to the oxygen-limiting stationary phase, a metabolic shift from aerobic respiration to mixed acid fermentation occurs for the reoxidation of the reductant NADH generated from central metabolic pathways. For *C. glutamicum*, l-lactate is a major fermentation end product produced by lactate dehydrogenase (LDH)-mediated pyruvate reduction [10]. In addition to lactate, acetate and succinate are produced by *C. glutamicum*. Acetate is produced from pyruvate by pyruvate quinone oxidoreductase or from acetyl-CoA by acetate kinase and phosphotransacetylase shunt as well as CoA transferase [15]. Succinate is produced from the anaplerotic reaction mediated by phosphoenolpyruvate carboxylase, followed by reductive TCA cycle reactions mediated by malate dehydrogenase, fumarase, and succinate dehydrogenase [10].

Expression analysis during the metabolic shift revealed the upregulation of the *gapA*-*pgk*-*tpi*-*ppc* operon encoding the glycolytic enzymes glyceraldehyde-3-phosphate dehydrogenase, phosphoglycerate kinase, and triosephosphate isomerase; the anaplerotic enzyme phosphoenolpyruvate carboxylase [10,16]. Consistent with the accumulation of organic acids, the *ldhA* and *mdh* genes encoding lactate and malate dehydrogenase, respectively, both of which are involved in the production of fermentation products, were upregulated [10,16]. By contrast, genes encoding the oxidative TCA cycle—including citrate synthase, aconitase, isocitrate dehydrogenase, and 2-oxoglutarate dehydrogenase—and ATP synthase were downregulated, indicating that aerobic respiration metabolism is suppressed under these conditions, whereas glycolytic metabolism is activated.

The *gapA* promoter of the *gapA* operon is regulated by multiple transcriptional regulators. SugR, a DeoR-type transcriptional regulator, represses *gapA* expression in the absence of sugar [17], where the glycolytic pathway is not essential. Sugar phosphates, including fructose-1-phosphate, glucose-6-phosphate, fructose-6-phosphate, and fructose-1,6-bisphosphate, that are generated via phosphotransferase system (PTS)-mediated sugar uptake and glycolysis negatively control the binding of SugR to the DNA, thereby relieving SugR-mediated repression [18,19,20,21]. The LuxR-type transcriptional regulator RamA positively regulates *gapA* expression [22], and its DNA binding is inhibited by reducing equivalent NAD(P)H, indicating that it is capable of sensing the cellular redox state [23]. GlxR, a cAMP receptor protein (CRP)-type transcriptional regulator, also positively regulates *gapA* expression [24]. Intracellular cAMP levels are controlled by the adenylate cyclase CyaB producing cAMP from ATP and the phosphodiesterase CpdA, degrading cAMP to AMP [25,26]. Although its physiological role remains elusive, the GntR-type regulator GntR1 binds to the *gapA* promoter in vivo and in vitro [22,27].

Transcriptional regulation of *ldhA* has also been extensively studied, and it has been shown that *ldhA* expression is repressed by SugR in the absence of sugar [18,20,21], similar to *gapA* expression. In addition, the FadR-type transcriptional regulator LldR represses *ldhA* expression [28]. It was first identified as a transcriptional repressor of the l-lactate utilization operon, which is induced in the presence of l-lactate [29]. LldR binding to target promoters is inhibited by l-lactate. SugR is the predominant repressor as *ldhA* is still repressed in the absence of sugar and the presence of l-lactate, under which LldR-mediated repression of the l-lactate utilization operon is alleviated. The binding site of SugR overlaps the −10 region of the *ldhA* promoter, and that of LldR overlaps the −35 region [28], consistent with the predominance of SugR over LldR.

**Figure 1:**
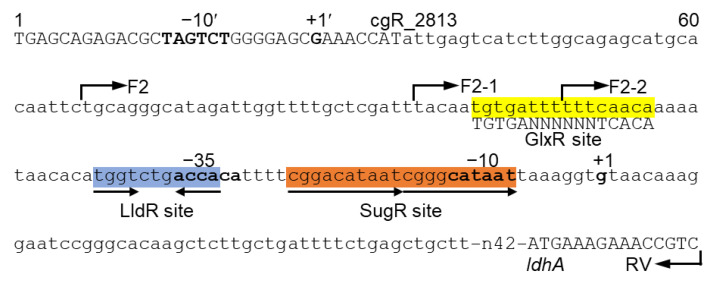
The *ldhA* promoter region and transcriptional regulator binding sites. The intergenic regions between *ldhA* and its upstream divergently transcribed gene cgR_2813 are indicated in lowercase, and the coding regions are indicated in uppercase. Bent arrows indicate the 5′ positions of the primers used for the amplification of the promoter region. The transcriptional start site (TSS) of *ldhA* and the −10 and −35 regions of the *ldhA* promoter are indicated with bold letters, and their positions with respect to the TSS are indicated in the sequence above. Transcription factor binding sites are highlighted with direct or inverted repeat sequences indicated by arrows. The putative alternative promoter with the TSS detected by RNA sequencing is indicated as +1′. The −10 region of the alternative promoter is indicated as −10′.

The *ldhA* promoter activity is suppressed at the exponential phase and upregulated during the transition to stationary phase [16]. This growth phase-dependent upregulation was observed even in the *sugR* and *lldR* double deletion mutant, in which *ldhA* expression is derepressed at the exponential phase [28]. This indicates the involvement of additional transcriptional regulators in *ldhA* expression. In silico and in vitro analyses have previously detected the binding site of the global regulator GlxR in the *ldhA* promoter region (Figure 1) [30]. This binding site is upstream of those of SugR and LldR. However, the physiological role of GlxR in the transcriptional regulation of *ldhA* expression has not yet been examined. In this study, we showed by mutational analysis of the GlxR-binding site that GlxR acts as a transcriptional activator of *ldhA* expression.

## 2. Materials and Methods

### 2.1. Bacterial Strains, Plasmids, and Culture Conditions

The bacterial strains and plasmids used in this study are listed in Table S1. For genetic manipulation, *Escherichia coli* strains were cultivated in lysogeny broth (LB) at 37 °C. *C. glutamicum* strains were routinely precultured at 33 °C overnight in nutrient-rich medium (A medium) [16] supplemented with 4% glucose. Cells were resuspended in fresh A medium and inoculated into 100 mL of A medium containing 1% glucose in a 500 mL flask. Growth was monitored by measuring the optical density (OD) at 610 nm. The antibiotic concentration used for *E. coli* cultures included 50 μg of ampicillin mL^−1^ and 50 μg of kanamycin mL^−1^, and for *C. glutamicum*, kanamycin (50 μg mL^−1^) was used.

### 2.2. Construction of Plasmids and Mutants

The oligonucleotides used in this study (Table S2) were obtained from FASMAC (Kanagawa, Japan). General recombinant experiments, including transformation of *E. coli* and plasmid extraction, were performed according to standard protocols [31]. To construct plasmids for the *ldhA* promoter–*lacZ* fusions, DNA fragments encompassing various lengths of the *ldhA* promoter region were amplified using the primers listed in Table S2 and cloned into the integration vector pCRA741 to form translational fusion with the *lacZ* gene on the vector. To modify the chromosomal GlxR-binding site, the binding site and both flanking regions were amplified by PCR (Table S2). The resulting two fragments were fused via overlapping PCR, digested with SalI, and cloned into the suicide vector pCRA725 [32]. Similarly, the flanking regions of the *atlR* gene were fused and cloned into pCRA725 to construct the plasmid pCRC664 for the deletion of the *atlR* gene. For efficient transformation of *C. glutamicum*, nonmethylated plasmids were obtained from *E. coli* JM110. *C. glutamicum* was transformed with the plasmids via electroporation and screened for kanamycin resistance in order to obtain the recombinants harboring chromosomal integration. To obtain marker-free mutants, the transformants were sequentially screened for sucrose resistance and kanamycin sensitivity as described previously [32]. The chromosomal mutations in the resulting mutants were confirmed by PCR amplification of the *ldhA* promoter region and direct sequencing of the PCR products using appropriate primers. To construct a plasmid for *glxR* overexpression, the *glxR* coding region was PCR amplified and cloned into the isopropyl-β-d-thiogalactopyranoside (IPTG)-inducible vector pCRB12iP [33]. The resulting plasmid was electroporated into both wild-type and mutant cells carrying the mutagenized GlxR-binding site within the *ldhA* promoter region. Overexpression of *glxR* was induced by supplementing the exponentially growing culture with 0.5 mM IPTG.

### 2.3. β-Galactosidase Assay

β-Galactosidase activity in *C. glutamicum* strains containing P*ldhA*–*lacZ* fusion was determined as described previously [16]. Briefly, cells permeabilized with toluene were incubated with *o*-nitrophenyl-β-galactosidase in Z buffer (60 mM Na_2_HPO_4_, 40 mM NaH_2_PO_4_, 10 mM KCl, 1 mM MgSO4, 0.28% (*v*/*v*) β-mercaptoethanol, which was added on use) at 30 °C. The absorbance values at 420 and 550 nm were monitored to calculate activity in Miller units as previously described [34]. We confirmed that a strain carrying the vacant vector pCRA741 containing the promoter-less *lacZ* had no LacZ activity.

### 2.4. Overexpression and Purification of His-Tagged GlxR and AtlR

Overexpression of His-tagged proteins was performed as described previously using the cold-inducible expression system [24]. Briefly, *E. coli* BL21(DE3) was transformed with pCRC620 or pCRC665 and grown at 37 °C in LB medium to an OD_600_ of 0.5. After cooling and incubation at 15 °C, 0.5 mM IPTG was added for induction of His-tagged protein, which was purified by affinity chromatography on Ni-NTA agarose (Qiagen, Hilden, Germany) according to the manufacturer’s instructions. The PD-10 column (GE Healthcare Bioscience, Piscataway, NJ, USA) was pre-equilibrated with buffer A (50 mM Tris-HCl, pH 7.5, 10 mM MgCl_2_, and 1 mM EDTA) to desalt the protein sample. The protein concentration was determined by Bio-Rad protein assay (Bio-Rad Laboratories Inc., Hercules, CA, USA) using bovine serum albumin as a standard.

### 2.5. Electrophoretic Mobility Shift Assay (EMSA)

EMSA was performed as described previously [24]. Briefly, Cy3-labeled DNA fragments used as probes in EMSA were generated by PCR using pCRC656, pCRC659, and pCRC660, containing the *ldhA* promoter region with or without the mutated GlxR-binding site as a template. GlxR was incubated with cAMP prior to incubation with the labeled probe. The resulting DNA–protein complexes were loaded onto a 5% polyacrylamide gel and visualized using a Typhoon TRIO Variable Mode Imager (GE Healthcare Bioscience).

### 2.6. RNA Extraction and Quantitative Reverse-Transcription Polymerase Chain (qRT-PCR)

For RNA stabilization, the sampled culture was mixed with an equal volume of RNAprotect bacterial reagent (Qiagen). After incubation at room temperature, cells were collected by centrifugation and stored at −80 °C. Total RNA was isolated using NucleoSpin^®^ RNA (MACHEREY-NAGEL, Düren, Germany) according to the manufacturer’s instructions. Purified RNA was further treated with DNaseI (Takara Bio Inc., Shiga, Japan), ethanol precipitated, and suspended in RNase-free water as described previously [35].

qRT-PCR was performed using the 7500 Fast Real-Time PCR System (Thermo Fisher Scientific, Waltham, MA, USA) and Power SYBR^®^ Green PCR Master Mix with MuLV Reverse Transcriptase and RNase Inhibitor from the GeneAmp RNA PCR Kit (Thermo Fisher Scientific) as described previously [17]. The primers listed in Table S2 were used. The comparative threshold cycle method (Thermo Fisher Scientific) was used to quantify relative expression, and the relative expression ratios of each gene were normalized using the values for 16S rRNA.

### 2.7. Analytical Method

Organic acids in culture supernatants were quantified using the Prominence 20A high-performance liquid chromatography (HPLC) system (Shimadzu, Kyoto, Japan) equipped with TSKgel OApack-A and OApack-P columns (Tosoh Corporation, Tokyo, Japan) and a photodiode array detector. All organic acids were detected at 210 nm. The flow rate of the mobile phase of 10% acetonitrile with 3.0 mM HClO_4_ was 0.8 mL/min, and the column temperature was set at 40 °C.

Glucose concentrations were determined using the enzyme electrode glucose sensor (BF-5; Oji Scientific Instruments, Hyogo, Japan).

## 3. Results

### 3.1. Involvement of the Putative GlxR-Binding Site in Transcription of ldhA

**Figure 2:**
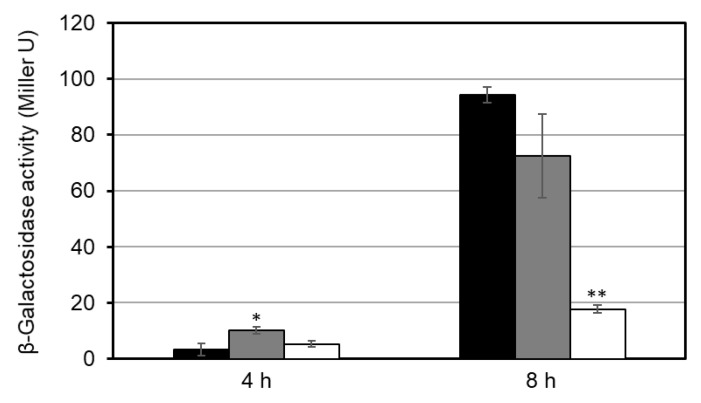
The promoter activity of *lacZ* fusions of a series of the *ldhA* promoter fragments. The *ldhA* promoter regions amplified with the primers F2/RV (black), F2-1/RV (gray), or F2-2/RV (white) were fused with *lacZ* and integrated into the wild-type chromosome. The resulting strains were grown in A medium supplemented with 1% glucose. β-Galactosidase activities of the cells at 4 h (i.e., exponential) and 8 h (i.e., stationary phase) were determined. Mean values obtained from three independent cultivations are shown with their standard deviations. *p*-values were calculated using an unpaired *t*-test. (*) *p* < 0.05; (**) *p* < 0.01 (comparison with F2/RV construct).

To examine whether the GlxR-binding site was involved in the transcriptional regulation of *ldhA* at the transition from the exponential to stationary phase, we constructed a series of P*ldhA*–*lacZ* fusion constructs. The constructs using primers F2 and F2-1 contained the intact GlxR-binding site, and the construct generated using the primer F2-2 lacked a portion of the site (Figure 1). The fusion constructs were individually integrated into the chromosome of the wild type. At the exponential phase (4 h) during cultivation in A medium supplemented with 1% glucose, the promoter activities of all of the fusion constructs were comparably low. Both F2 and F2-1 fusions exhibited an increase in promoter activity at the stationary phase (8 h) (Figure 2). By contrast, the increase in the activity of the F2-2 promoter fusion at the stationary phase was much smaller than that of the other fusions (Figure 2). This indicates that the GlxR-binding site is involved in the *ldhA* upregulation at the onset of the stationary phase and that GlxR functions as a transcriptional activator.

**Figure 3:**
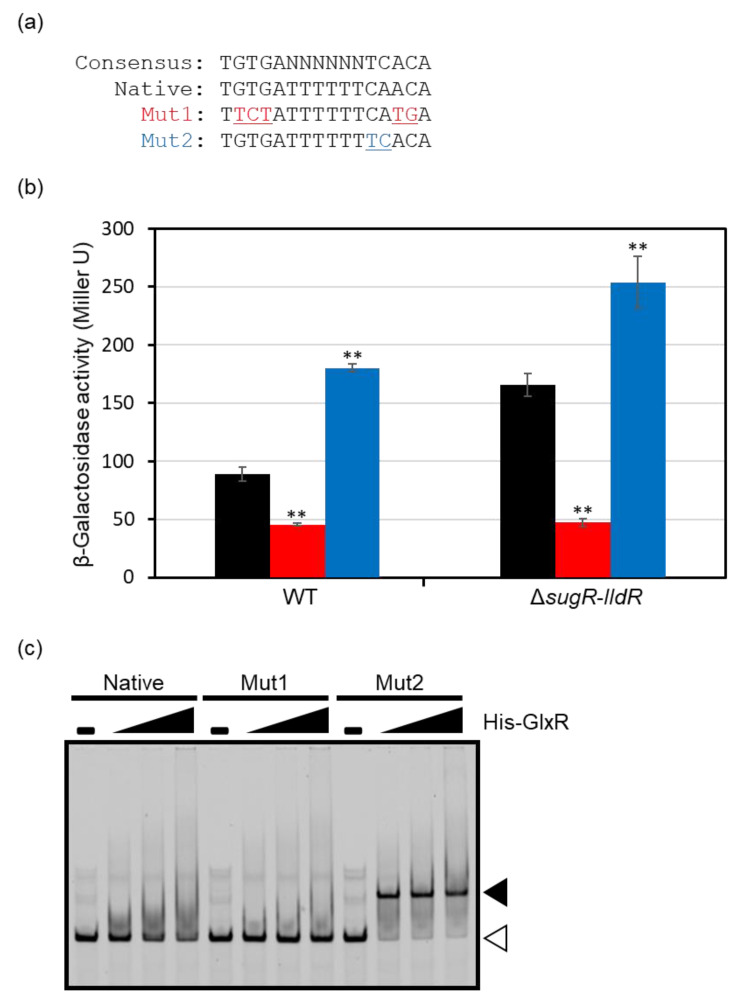
Effects of mutations in the GlxR-binding site. (**a**) Mutations introduced into the GlxR-binding site in the *ldhA* promoter. Consensus binding site (top) is shown for reference. The mutation mut1 was designed to exchange highly conserved nucleotides, and the mutation mut2 was designed to create the consensus site. (**b**) Promoter activity of the *lacZ* fusions with the *ldhA* promoter carrying the native (black), mut1 (red), or mut2 (blue) GlxR-binding site in either the wild type or *sugR*–*lldR* double deletion mutant. Overnight culture of each strain in A medium supplemented with 4% glucose was used for the LacZ assay. Mean values with standard deviations from more than four clones are shown. *p*-values were calculated using an unpaired *t*-test. (**) *p* < 0.01 (comparison with the native constructs). (**c**) Electrophoretic mobility shift assay using His-tagged GlxR. The Cy3-labeled probes (10 nM) encompassing the *ldhA* promoter region used for the construction of the *lacZ* fusions were incubated with varying amounts of His-tagged GlxR: 0.5, 1.0, and 2.0 μM. Free DNA probe and DNA–protein complex are indicated with white and black arrowheads. The probes contain nonspecific bands.

To investigate the importance of the GlxR-binding site for *ldhA* promoter activity, we introduced mutations into the site upon the promoter–*lacZ* fusion. We modified the GlxR-binding site to inhibit (mut1) and enhance the binding of GlxR (mut2) by exchanging nucleotides at the conserved position in the binding site (Figure 3a). The *lacZ* fusions were individually integrated into the chromosome of the wild type. The F2 promoter–*lacZ* fusion carrying the mutation mut1 exhibited lower activity than the native one (Figure 3b). By contrast, the F2 promoter–*lacZ* fusion carrying the mutation mut2 showed higher activity than that of the native promoter fusion. The same fusion constructs were also integrated into the chromosome of the *sugR*–*lldR* double deletion mutant, in which the *ldhA* promoter was derepressed. As observed in the wild-type background, the mutation mut1 reduced promoter activity, and the mutation mut2 increased activity in the *sugR*–*lldR* deletion mutant background (Figure 3b). These findings indicate that GlxR acts as a transcriptional activator by binding to the site, and its function is independent of the known repressors.

The effects of the mutations on GlxR binding were investigated using EMSA (Figure 3c). After incubation with GlxR and cAMP, the same DNA probe encompassing the *ldhA* promoter region as was used for the F2 promoter–*lacZ* fusion construct yielded a smeared band shift on the gel, indicating the interaction of GlxR with the *ldhA* promoter. The DNA fragment carrying the mutation mut1 showed no shifted band. Incubation with the DNA fragment carrying the mutation mut2, in contrast, resulted in a much clearer band shift than the native one. This demonstrated that the mutations mut1 and mut2 inhibited and enhanced GlxR binding, respectively, as expected.

### 3.2. Effects of Chromosomal Mutations in the GlxR-Binding Site in the ldhA Promoter

**Figure 4:**
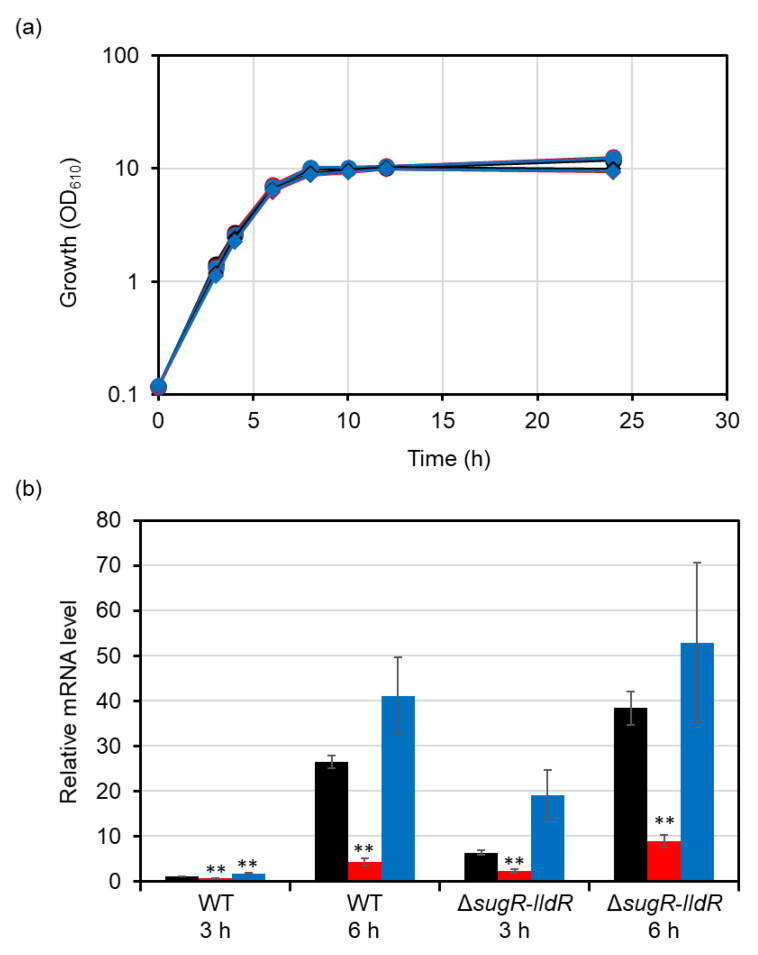
Effects of mutations in the GlxR-binding site on *ldhA* expression. (**a**) Growth of the wild type (circles) and the *sugR*–*lldR* deletion mutant (diamonds) with the native (black), mut1 (red), or mut2 (blue) GlxR-binding site in the *ldhA* promoter in A medium supplemented with 1% glucose. RNA was extracted from bacterial culture samples at 3 h and 6 h. (**b**) Transcript levels of the *ldhA* gene in the wild type and the *sugR*–*lldR* deletion mutant with the native (black), mut1 (red), or mut2 (blue) GlxR-binding site in the *ldhA* promoter were determined by quantitative reverse-transcription polymerase chain reaction analysis. The transcript level in the wild type with the native promoter at 3 h was standardized to 1. Mean values obtained from three independent cultivations are shown with their standard deviations. *p*-values were calculated using an unpaired *t*-test (**) *p* < 0.01.

The GlxR regulon consists of more than 200 genes. Probably because of its large impact on the transcriptome, the *glxR* deletion mutant shows severely retarded growth compared with the wild type [24]. This prevents the investigation of the physiological function of GlxR using the knockout mutant. To directly examine the GlxR role in the regulation of *ldhA*, we introduced the same mutations (i.e., mut1 and mut2) into the binding site on the chromosome and investigated their effects on *ldhA* expression. The chromosomal mutations had no effect on growth (Figure 4a). As we have shown that *ldhA* expression is highly upregulated at 6 h compared with that at 3 h [21], RNA was extracted at these time points. The mutant carrying the mutation mut1 in the GlxR-binding site exhibited lower expression of *ldhA* than the wild type at both the exponential (3 h) and transition (6 h) phases (Figure 4b). By contrast, the presence of mut2 in the GlxR-binding site showed apparently but not significantly increased expression of *ldhA* compared with the wild type at the transition phase (*p* = 0.097, 6 h). Thus, the GlxR-binding site was required for the upregulation of *ldhA* at the stationary phase. The same mutations were introduced into the chromosome of the *sugR*–*lldR* deletion mutant. As *ldhA* expression is fully derepressed in this mutant, *ldhA* expression can be observed even at the exponential phase. As observed in the wild type, the mutation mut1 reduced *ldhA* expression, whereas mut2 enhanced it (*p* = 0.054, 3 h) (Figure 4b). This result showed that GlxR activated *ldhA* expression at the exponential phase as well as at the stationary phase.

### 3.3. Effects of the Deletion of the cyaB Gene Encoding Adenylate Cyclase

GlxR belongs to a CRP-type transcriptional regulator family and binds to target binding sites in a cAMP-dependent manner in vitro [30,36,37]. In the *C. glutamicum* genome, a sole adenylate cyclase is encoded by *cyaB* [25,38]. To examine the effect of cAMP deficiency on *ldhA* expression, a *cyaB* deletion mutant was constructed, which grew slightly slower than the wild type (Figure S1a). The *ldhA* expression level in the *cyaB* mutant was comparable to that in the wild type at the exponential and transition phases (Figure S1b), indicating that the upregulation observed at the transition phase does not require cAMP. The *aceA* gene encoding isocitrate lyase in the glyoxylate shunt has been shown to be repressed by GlxR and affected by intracellular cAMP levels [26,37]. The expression of *aceA* in the *cyaB* deletion mutant was higher than that in the wild type (Figure S1b), confirming deregulation of GlxR in the mutant. We also deleted the *cyaB* gene in the background of the *sugR* deletion mutant. The growth of the *cyaB*–*sugR* deletion mutant was slower than that of the wild type and transiently stalled at 12 h of cultivation, but the OD at 24 h reached the level of the wild type (Figure S1a). The *ldhA* expression in the mutant was higher than that in either the wild type or *sugR*–*lldR* double deletion mutant, in which *ldhA* expression is derepressed, at the exponential phase (3 h) (Figure S1b). However, *ldhA* expression in the *cyaB*–*sugR* double mutant was lower than that in the *sugR*–*lldR* mutant at the transition phase (6 h). The combination of the *sugR* deletion with cAMP deficiency by deletion of *cyaB* likely caused pleiotropic effects on gene expression. This was possibly reflected by the observation that the expression of the *aceA* gene, derepressed in the *cyaB* single deletion mutant, was lowered in the *cyaB*–*sugR* deletion mutant compared to that in the wild type.

### 3.4. Effects of the Overexpression of GlxR on ldhA Expression

To examine the effects of *glxR* overexpression on the expression of *ldhA*, the plasmid containing *glxR* under control of the IPTG-inducible *tac* promoter was constructed. The plasmid was introduced into both the wild type and mutants containing mutations (mut1 and mut2) in the GlxR-binding site. The *glxR* gene was induced with 0.5 mM IPTG, and the expression levels of *ldhA* were determined via qRT-PCR. Before IPTG supplementation, *ldhA* expression in the mut1 and mut2 strains was lower and higher than that in the wild type, respectively. The expression of *glxR* was, as expected, increased by approximately 20-fold following 15 min of IPTG supplementation. However, the overexpression of *glxR* had almost no effect on the expression of *ldhA* in the wild type (data not shown).

**Figure 5:**
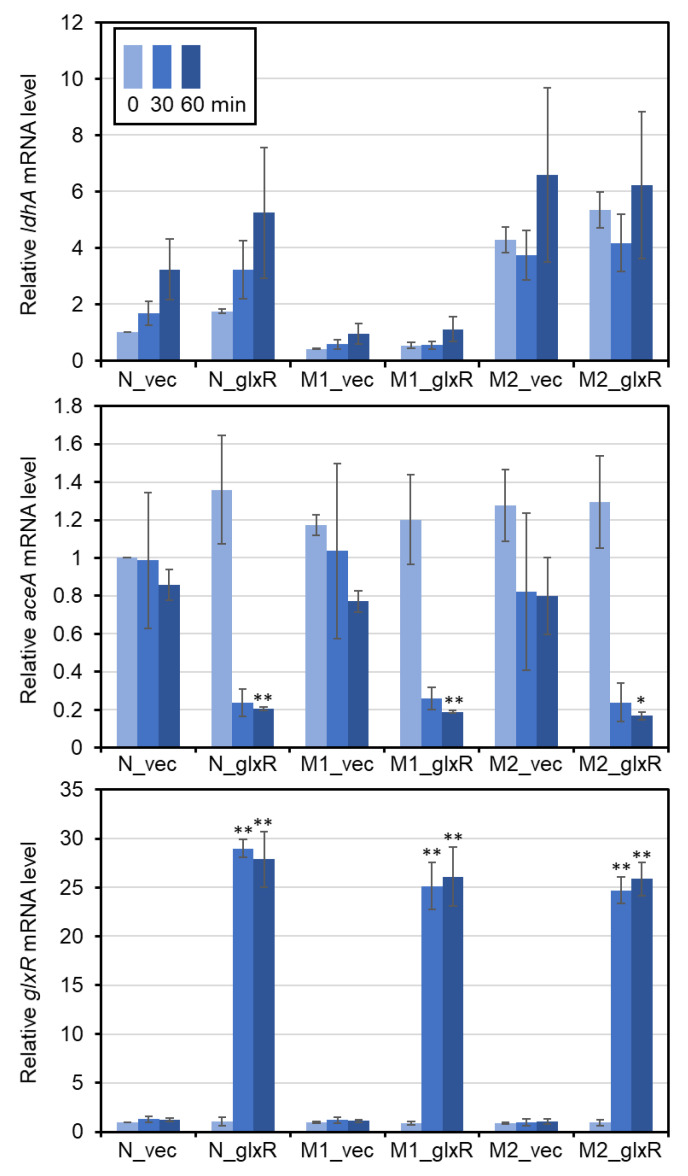
Overexpression of *glxR* elicited a positive effect on *ldhA* expression. The *sugR*–*lldR* deletion mutants harboring the native (N), mut1 (M1), or mut2 (M2) GlxR-binding site in the *ldhA* promoter were transformed with either the control vector pCRB12iP (vec) or the plasmid pCRC663 for *glxR* overexpression (glxR) and were induced with 0.5 mM isopropyl β-d-1-thiogalactopyranoside for 30 and 60 min at the exponential growth phase (3 h from the beginning of cultivation). The transcript levels of the *ldhA* (top), *aceA* (middle), and *glxR* (bottom) genes were determined by quantitative reverse-transcription polymerase chain reaction analysis. Transcript levels in the wild type carrying the vector before induction were standardized to 1. Mean values obtained from three independent cultivations are shown with their standard deviations. *p*-values were calculated using an unpaired *t*-test. (*) *p* < 0.05; (**) *p* < 0.01 (comparison of *glxR* overexpression strains with vector strains at the same sampling points).

The *glxR* overexpression plasmid was introduced into the *sugR*–*lldR* deletion mutant as well as the strain containing the mutated GlxR-binding site (mut1 and mut2) on the chromosome. After 30 min of IPTG supplementation, *ldhA* expression under the control of the native promoter in the *sugR*–*lldR* mutant was apparently but not significantly upregulated compared with the control strain carrying the blank vector (*p* = 0.11, Figure 5). In the mutants with the mutated binding site (mut1 and mut2), the overexpression of *glxR* had no effect on *ldhA* expression. Whereas GlxR was unable to bind to the *ldhA* promoter and affect *ldhA* expression in the mut1 strain, the wild-type level of GlxR was likely high enough for the activation of *ldhA* expression in the mut2 strain. In contrast to the deletion of the *cyaB* gene, the overexpression of *glxR* resulted in the reduction of *aceA* expression, confirming that the regulation mediated by GlxR was promoted by IPTG-induced overexpression.

### 3.5. Effects of Chromosomal Mutations in the GlxR-Binding Site on Organic Acid Profiles

**Figure 6:**
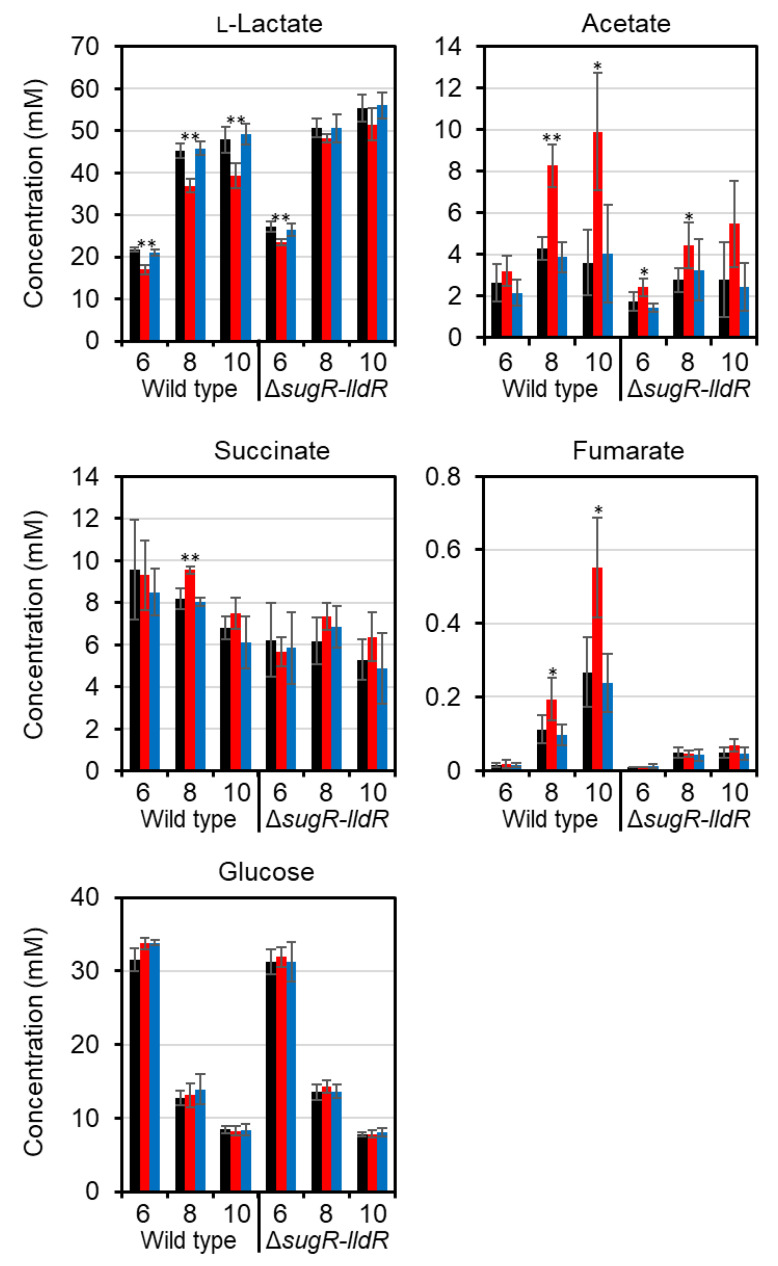
Organic acids and glucose in culture supernatants. The wild type and *sugR*–*lldR* deletion mutant with the native (black), mut1 (red), or mut2 (blue) GlxR-binding site in the *ldhA* promoter were grown in A medium supplemented with 1% glucose. The culture supernatants at 6, 8, and 10 h were analyzed. Mean values obtained from at least three independent cultivations are shown with their standard deviations. *p*-values were calculated using an unpaired *t*-test. (*) *p* < 0.05; (**) *p* < 0.01 (comparison with the parental wild type).

The mutations in the GlxR-binding site in the *ldhA* promoter region altered *ldhA* expression. The effects of this modification of the expression on organic acid excretion were investigated. Organic acids in the culture supernatant from the transition (6 h) to stationary (10 h) phases were quantified by HPLC. At the exponential phase (4 h), few organic acids were produced. The mutant with the mutations mut1, in which *ldhA* expression was suppressed, excreted a significantly lower amount of lactate than the wild type (Figure 6). The consumption of glucose was comparable for all strains. In the *sugR*–*lldR* deletion mutant background, in which the repression of *ldhA* was relieved, the mut1 mutation similarly lowered the amount of excreted lactate, but the effects were smaller than those in the wild-type background and the difference was not significant after 8 h. The reduced lactate production in the mut1 promoter mutant was likely compensated for by the increased production of other organic acids—succinate, acetate, and fumarate—compared with that produced by the native promoter strains. This is consistent with the fact that the excretion levels of these organic acids by the *sugR*–*lldR* deletion mutant derivatives were lower than those by the wild-type derivatives. The mutants with the mutations mut2 excreted lactate in an amount comparable to that of the native promoter strains. At the sampling points in this study, the increase in *ldhA* expression in the mut2 mutants had no effect on organic acid production, suggesting that the LDH expression was not rate limiting for l-lactate production in the wild type and *sugR*–*lldR* deletion mutant background.

## 4. Discussion

In this study, we showed that the key fermentative gene *ldhA* is directly activated by GlxR. GlxR was not obtained by previous DNA affinity purification using the *ldhA* promoter region as a ligand [20,21], which identified the two repressors SugR and LldR. This is probably because the binding site found in the *ldhA* promoter region incompletely matches the consensus binding motif: 5′-TGTGA-N6-TCACA-3′. Indeed, the shifted band observed in EMSA was smeared, although it was cAMP dependent (Figure 3c). The similar smeared shifted bands have been observed in previous studies on GlxR [30,36]. The mutations of nucleotides at the conserved position in the binding motif (mut1) completely inhibited GlxR binding and reduced *ldhA* expression. Previous studies have similarly introduced the mismatch mutations in the GlxR-binding site to investigate the regulatory role of GlxR [24,26,39,40]. By contrast, in this study, the exchanging of two nucleotides to match the conserved motif (mut2: TGTGATTTTTTCAACA –> TGTGATTTTTTTCACA) drastically improved GlxR binding, as shown by the clear shifted band. The same mutations increased *ldhA* promoter activity and *ldhA* expression. Thus, to the best of our knowledge, this is the first case of a GlxR regulon, whose expression was shown to be modified by the affinity of the target binding site.

The mutations of the GlxR-binding site in the *ldhA* promoter may affect the binding of other transcription factors, resulting in the alteration of *ldhA* expression. Previous DNA affinity purification using the *ldhA* promoter region as a ligand [20,21] identified AtlR, a DeoR-type transcriptional regulator functioning as a repressor of the expression of the arabitol/mannitol catabolic genes [41,42]. Our EMSA using the recombinant AtlR showed that binding of AtlR to the *ldhA* promoter region was slightly inhibited by the mut1 mutation in vitro, but not affected by mut2 (Figure S2a). We constructed the *atlR* deletion mutant based on the *sugR*–*lldR* double deletion mutants carrying the native, mut1, or mut2 GlxR-binding site in the *ldhA* promoter and assessed the effects of the deletion on *ldhA* expression. The *atlR* deletion had no effect on *ldhA* expression in any genetic background, indicating that the mutations mut1 and mut2 had no effect on AtlR function, if any, in *ldhA* regulation (Figure S2b). A previous study also showed that single deletion of *atlR* had no effect on *ldhA* expression [20].

During growth on acetate, expression of *ldhA* is repressed by SugR. Moreover, as intracellular cAMP levels during growth on acetate are lower than those on glucose [25], GlxR-mediated activation of *ldhA* would be alleviated to save cellular resources. Similarly, the glycolytic genes *pfkA* encoding phosphofructokinase and the *gapA*-*pgk*-*tpi*-*ppc* operon encoding glyceraldehyde-3-phosphate dehydrogenase, phosphoglycerate kinase, triose phosphate isomerase, and phosphoenolpyruvate carboxylase were regulated by both SugR and GlxR [17,24,43]. These two global regulators coordinately control the glycolytic pathway and redox balance. In other bacteria, lactate dehydrogenase expression is important during anaerobic metabolism and is regulated by redox-sensing transcription factors [44,45,46]. How the intracellular cAMP levels of *C. glutamicum* are controlled in response to environmental changes merits further investigation for the understanding of the global regulatory role of GlxR.

Even in strains containing the mut1 mutations, *ldhA* expression showed more than a threefold increase at the transition phase (Figure 4). The group 2 sigma factor SigB is likely responsible for this upregulation. We have previously shown that deletion of *sigB* reduced *ldhA* expression by twofold [47]. Comprehensive transcriptional start sites (TSSs) determined by RNA-seq revealed another site upstream of the TSS previously identified (shown as +1′ in Figure 1) [48,49]. This whole TSS determination by RNA-seq also found characteristic promoter structures. Over 70% of SigB-dependent promoters contain a G at position −5, two nucleotides downstream of the conserved −10 region, with respect to the TSS [49]. Although the *ldhA* promoter investigated in this study does not contain a G at this position, the upstream novel *ldhA* promoter does. Therefore, the novel promoter likely functions as the SigB-dependent promoter and may upregulate *ldhA* expression at the transition phase. SigB also controls the glycolytic genes under aerobic growth conditions and oxygen deprivation [47]. Investigation of the function of the upstream promoter and involvement of SigB in transcription from the promoter will provide further insights into the regulatory mechanism of fermentative genes in *C. glutamicum*.

## Supplementary Materials

The following are available online at https://www.mdpi.com/2076-2607/9/3/550/s1, Figure S1: Effects of *cyaB* gene deletion on *ldhA* expression, Figure S2: Effects of the *atlR* gene deletion on *ldhA* expression, Table S1: Bacterial strains and plasmids used in this study, Table S2: oligonucleotide primers used in this study.
