# Supplementary material for: The ldhA Gene Encoding Fermentative l-Lactate Dehydrogenase in Corynebacterium Glutamicum Is Positively Regulated by the Global Regulator GlxR
Source: Microorganisms. 2021 Mar 6;9(3):550. doi: 10.3390/microorganisms9030550 (PMC7999487; doi:10.3390/microorganisms9030550)
Supplement: Supplementary file 1 [file microorganisms-09-00550-s001.zip › Supplementary text1_final.docx]

The caption of supplementary figures.

**Figure S1.** Effects of *cyaB* gene deletion on *ldhA* expression. (a) Growth of the wild type (blue circles), the deletion mutants of *cyaB* (orange triangles), and *cyaB*–*sugR* (green squares) in A medium supplemented with 1% glucose. RNA was extracted from bacterial culture samples at 3 h and 6 h. (b) Expression of *ldhA* and *aceA* in the wild type (gray), the deletion mutants of *cyaB* (orange), *cyaB*–*sugR* (yellow), and *sugR*–*lldR* (green). Gene transcript levels were determined by quantitative reverse-transcription polymerase chain reaction analysis. Transcript levels in the wild type at 3 h were standardized to 1. Mean values obtained from three independent cultivations are shown with their standard deviations. *P*-values calculated using an unpaired *t*-test were shown. (*) *P* < 0.05; (**) *P* < 0.01. (comparison with the wild type)

**Figure S2.** Effects of the *atlR* gene deletion on *ldhA* expression. (a) Electrophoretic mobility shift assay using His-tagged *atlR*. The Cy3-labeled probes (10 nM) encompassing the *ldhA* promoter region used for the construction of the *lacZ* fusions were incubated with varying amounts of His-tagged AtlR: 1.0, 2.0, and 4.0 μM. The probes contain nonspecific bands. (b) The wild type (gray) and the deletion mutants of *atlR* (orange), *atlR*–*sugR* (yellow), *sugR*–*lldR* (green), *atlR*–*sugR*–*lldR* with the native *ldhA* promoter (black), *atlR*–*sugR*–*lldR* with the mut1 GlxR binding site (red), and *atlR*–*sugR*–*lldR* with the mut2 binding site (blue) were grown in A medium supplemented with 1% glucose. RNA was extracted from bacterial culture samples at 3 and 6 h. The transcript levels of the *ldhA* gene were determined by quantitative reverse-transcription polymerase chain reaction analysis. The transcript level in the wild type at 3 h was standardized to 1. Mean values obtained from three independent cultivations are shown with their standard deviations. For the *sugR*–*lldR* deletion mutants, values derived within Figure 4 were used to create the bar graph. *P*-values calculated using an unpaired *t*-test were shown. (**) *P* < 0.01.
