# Supplementary material for: The ldhA Gene Encoding Fermentative l-Lactate Dehydrogenase in Corynebacterium Glutamicum Is Positively Regulated by the Global Regulator GlxR
Source: Microorganisms. 2021 Mar 6;9(3):550. doi: 10.3390/microorganisms9030550 (PMC7999487; doi:10.3390/microorganisms9030550)
Supplement: Supplementary file 1 [file microorganisms-09-00550-s001.zip › Table S1.docx]

Table S1. Bacterial strains and plasmids used in this study

| Strain or plasmid | Relevant characteristics | Source or reference |
| --- | --- | --- |
| Strains |  |  |
| *E. coli* |  |  |
| JM109 | *recA1 endA1 gyrA96 thi hsdR17 supE44 relA1* Δ(*lac-proAB*)/F’[*traD36 proAB*^+^ *lacI^q^ lacZ*ΔM15] | Takara |
| JM110 | *dam dcm supE44 hsdR17 tih leu rpsL lacy galK galT ara tonA thr tsx* Δ(*lac-proAB*)/F’[*traD36 proAB^+^ lacI^q^ lacZ*ΔM15] | [[1](#_ENREF_1)] |
| BL21(DE3) | F^–^ *ompT* *gal* *dcm* *lon* *hsdS*_B_(r_B_^-^ m_B_^-^) λ(DE3) | [[2](#_ENREF_2)] |
| *C. glutamicum* |  |  |
| R (JCM 18229) | Wild-type strain | [[3](#_ENREF_3)] |
| KT21 | R with deletion in *sugR* and *lldR* | [[4](#_ENREF_4)] |
| TN06 | R with deletion in *cyaB* | [[5](#_ENREF_5)] |
| KT45 | R with the P*ldhA*-*lacZ* fusion in pCRC656 | This study |
| KT46 | R with the P*ldhA*-*lacZ* fusion in pCRC657 | This study |
| KT47 | R with the P*ldhA*-*lacZ* fusion in pCRC658 | This study |
| KT48 | R with the P*ldhA*-*lacZ* fusion in pCRC659 | This study |
| KT49 | R with the P*ldhA*-*lacZ* fusion in pCRC660 | This study |
| KT50 | KT21 with the P*ldhA*-*lacZ* fusion in pCRC656 | This study |
| KT51 | KT21 with the Pl*dhA*-*lacZ* fusion in pCRC659 | This study |
| KT52 | KT21 with the P*ldhA*-*lacZ* fusion in pCRC660 | This study |
| KT53 | R with the mutated (mut1) GlxR binding site in the *ldhA* promoter region | This study |
| KT54 | R with the mutated (mut2) GlxR binding site in the *ldhA* promoter region | This study |
| KT55 | KT21 with the mutated (mut1) GlxR binding site in the *ldhA* promoter region | This study |
| KT56 | KT21 with the mutated (mut2) GlxR binding site in the *ldhA* promoter region | This study |
| KT57 | TN06 with deletion in *sugR* | This study |
| KT58 | R with deletion in *atlR* | This study |
| KT59 | KT58 with deletion in *sugR* | This study |
| KT60 | KT21 with deletion in *atlR* | This study |
| KT61 | KT55 with deletion in *atlR* | This study |
| KT62 | KT56 with deletion in *atlR* | This study |
| Plasmids |  |  |
| pCold | Apr: a vector for cold-inducible expression | TaKaRa |
| pCRA725 | Km^r^; the suicide vector containing the *B. subtilis* *sacB* gene | [[6](#_ENREF_6)] |
| pCRB12iP | Km^r^; *lacI*^q^, P*tac*, IPTG-inducible vector | [[7](#_ENREF_7)] |
| pCRA741 | Km^r^; pCRA725 with a 2.0-kb PCR fragment from strain-specific island 7 and a 3.1-kb PCR fragment containing the *E. coli* *lacZ* gene | [[8](#_ENREF_8)] |
| pCRC620 | Ap^r^: pColdI with a coding region of the *glxR* gene | [[9](#_ENREF_9)] |
| pCRC656 | Km^r^; pCRA741 with the *ldhA* promoter fragment amplified with PldhAFW2 and PldhARVNaeI | This study |
| pCRC657 | Km^r^; pCRA741 with the *ldhA* promoter fragment amplified with PldhAFW2-1 and PldhARVNaeI | This study |
| pCRC658 | Km^r^; pCRA741 with the *ldhA* promoter fragment amplified with PldhAFW2-2 and PldhARVNaeI | This study |
| pCRC659 | Km^r^; pCRA741 with the same *ldhA* promoter fragment as in pCRC656 with the mutated GlxR binding site (mut1) | This study |
| pCRC660 | Km^r^; pCRA741 with the same *ldhA* promoter fragment as in pCRC656 with the mutated GlxR binding site (mut2) | This study |
| pCRC661 | Km^r^; pCRA725 with a fragment containing the *ldhA* promoter with the mutated GlxR binding site (mut1) for introduction of the mutation | This study |
| pCRC662 | Km^r^; pCRA725 with a fragment containing the *ldhA* promoter with the mutated GlxR binding site (mut2) for introduction of the mutation | This study |
| pCRC663 | Km^r^; pCRB12iP with a fragment containing the *glxR* gene for IPTG-inducible overexpression | This study |
| pCRC664 | Km^r^; pCRA725 with a fragment containing the upstream and downstream regions of the *atlR* gene for deletion | This study |
| pCRC665 | Ap^r^: pColdI with a coding region of the *atlR* gene | This study |

1. Sambrook, J.; Fritsch, E. F.; Maniatis, T., *Molecular cloning: a Laboratory Manual, 2nd edn.* Cold Spring Harbor, NY: Cold Spring Harbor Laboratory, 1989.

2. Studier, F. W.; Moffatt, B. A., Use of bacteriophage T7 RNA polymerase to direct selective high-level expression of cloned genes. *J Mol Biol* **1986,** *189*, 113-130.

3. Yukawa, H.; Omumasaba, C. A.; Nonaka, H.; Kós, P.; Okai, N.; Suzuki, N.; Suda, M.; Tsuge, Y.; Watanabe, J.; Ikeda, Y.; Vertès, A. A.; Inui, M., Comparative analysis of the *Corynebacterium glutamicum* group and complete genome sequence of strain R. *Microbiology* **2007,** *153*, 1042-1058.

4. Toyoda, K.; Teramoto, H.; Inui, M.; Yukawa, H., The *ldhA* gene, encoding fermentative L-lactate dehydrogenase of *Corynebacterium glutamicum*, is under the control of positive feedback regulation mediated by LldR. *J. Bacteriol.* **2009,** *191*, 4251-4258.

5. Nishimura, T.; Teramoto, H.; Toyoda, K.; Inui, M.; Yukawa, H., Regulation of the nitrate reductase operon *narKGHJI* by the cAMP-dependent regulator GlxR in *Corynebacterium glutamicum*. *Microbiology* **2011,** *157*, 21-28.

6. Inui, M.; Murakami, S.; Okino, S.; Kawaguchi, H.; Vertès, A. A.; Yukawa, H., Metabolic analysis of *Corynebacterium glutamicum* during lactate and succinate productions under oxygen deprivation conditions. *J. Mol. Microbiol. Biotechnol.* **2004,** *7*, 182-196.

7. Toyoda, K.; Teramoto, H.; Gunji, W.; Inui, M.; Yukawa, H., Involvement of regulatory interactions among global regulators GlxR, SugR, and RamA in expression of *ramA* in *Corynebacterium glutamicum*. *J. Bacteriol.* **2013,** *195*, 1718-1726.

8. Inui, M.; Suda, M.; Okino, S.; Nonaka, H.; Puskás, L. G.; Vertès, A. A.; Yukawa, H., Transcriptional profiling of *Corynebacterium glutamicum* metabolism during organic acid production under oxygen deprivation conditions. *Microbiology* **2007,** *153*, 2491-2504.

9. Toyoda, K.; Teramoto, H.; Inui, M.; Yukawa, H., Genome-wide identification of *in vivo* binding sites of GlxR, a cyclic AMP receptor protein-type regulator in *Corynebacterium glutamicum*. *J. Bacteriol.* **2011,** *193*, 4123-4133.
