# Supplementary material for: The ldhA Gene Encoding Fermentative l-Lactate Dehydrogenase in Corynebacterium Glutamicum Is Positively Regulated by the Global Regulator GlxR
Source: Microorganisms. 2021 Mar 6;9(3):550. doi: 10.3390/microorganisms9030550 (PMC7999487; doi:10.3390/microorganisms9030550)
Supplement: Supplementary file 1 [file microorganisms-09-00550-s001.zip › Table S2.docx]

Table S2. Oligonucleotide primers used in this study

| Primer name | Sequence (5ʹ-3ʹ) | Note |
| --- | --- | --- |
| P*ldhA*-*lacZ* fusions and mutant strains construction | | |
| PldhAFW2 | TGCAGGGCATAGATTGGTTTTGC |  |
| PldhAFW2-1 | TACAATGTGATTTTTTCAACAAAAATAAC |  |
| PldhAFW2-2 | TTTCAACAAAAATAACACATGG |  |
| PldhARVNaeI | ATTGCCGGCGGTTTCTTTCATTTTCGATC |  |
| PldhAmutFW | AATTCTATTTTTTCGGACAAAATAACACATGGTCTGAC | For mut1 in GlxR site |
| PldhAmutRV | TTGTCCGAAAAAATAGAATTGTAAATCGAGCAAAACC | For mut1 in GlxR site |
| PldhAmut2FW | GTGATTTTTTTCACAAAAATAACACATGGTCTGAC | For mut2 in GlxR site |
| PldhAmut2RV | GTTATTTTTGTGAAAAAAATCACATTGTAAATC | For mut2 in GlxR site |
| ldhA_FW_SalI2 | TTTTTCAGTCGACCACGGGCTACCCGAACG |  |
| ldhA_RV_Sal2 | GCAGTGCGTCGACTGGGACTGCAACGTCTTGG |  |
| cgR_0187FW_xba | ACTCTAGATCGTCAGTGAAAAATGATGG | For atlR deletion |
| cgR_0187RV_xba | GTTCTAGAACCACAATCTCATGGCCGAAAATC | For atlR deletion |
| cgR_0187invFW | CCAAGTGATTGAAGAGGATTTTTAAGATGGCTTTGG | For atlR deletion |
| cgR_0187invRV | CCTCTTCAATCACTTGGGACATGTGAAAAAGTTAC | For atlR deletion |
| qRT-PCR | | |
| 16SFW_RT | TCGATGCAACGCGAAGAAC |  |
| 16SRV_RT | GAACCGACCACAAGGGAAAAC |  |
| gapA_RT_FW | AGGTGCCAAGAAGGTCATCATC |  |
| gapA_RT_RV | GACTCGTGGTTCACACCGTAAAC |  |
| ldhA_RT_FW | GAACACGGCGACACTGAACTT |  |
| ldhA_RT_RV | AGCATGCGGCTAAGCGATA |  |
| glxR_RT_FW | CACGCGGAGCAACAATCTT |  |
| glxR_RT_RV | TGGCGTGCAAGCTTCACTT |  |
| aceA_RT_FW | TCCTTCGTTGACCTGCAGAAC |  |
| aceA_RT_RV | AACCTCACGCTGGTGCTTAAC |  |
| Overexpression of GlxR | | |
| cgR_0377_FWKpnI_2 | TCGGTACCACATTAATAGATGCCTCCAG |  |
| cgR_0377_RVKpnI | TTGGTACCTTATCGAGCGCGACGTGCCAAATG |  |
| EMSA | | |
| GlxRHisFWNde | GAGAAAACATATGGAAGGTGTACAGGAGATCC | For recombinant GlxR |
| GlxRHisRVSal | GCGCTTGTCGACTTATCGAGCGCGACGTGCCAAATG | For recombinant GlxR |
| cgR_0187_FW_Nde | ACTTTTTCATATGTCCCAAGTGATTCCCGCCAGC | For recombinant AtlR |
| cgR_0187_RV_Xba | AATCTAGATTAAAAATCCTCTTCCGTTGGGGTATCC | For recombinant AtlR |
| LlacZind_RVcy3 | Cy3-CACGACGTTGTAAAACGACGGGATC | For probe preparation |
