# Supplementary figures and images for: The ldhA Gene Encoding Fermentative l-Lactate Dehydrogenase in Corynebacterium Glutamicum Is Positively Regulated by the Global Regulator GlxR
Source: Microorganisms. 2021 Mar 6;9(3):550. doi: 10.3390/microorganisms9030550 (PMC7999487; doi:10.3390/microorganisms9030550)

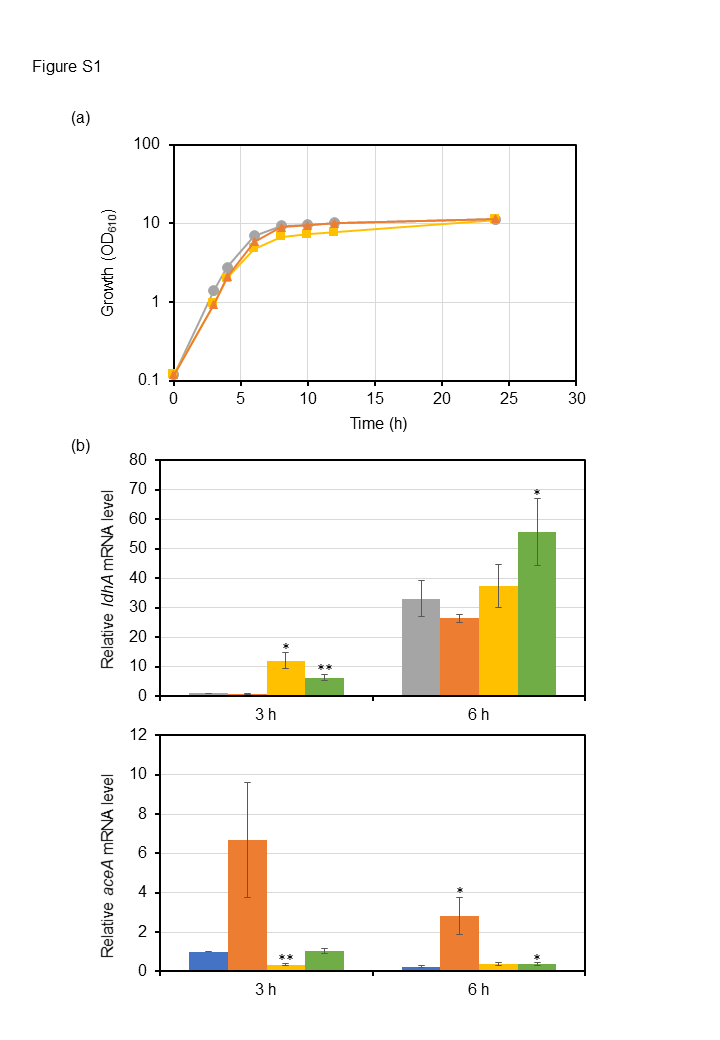

Supplement: Supplementary file 1 [file microorganisms-09-00550-s001.zip › Figure S1.TIF]

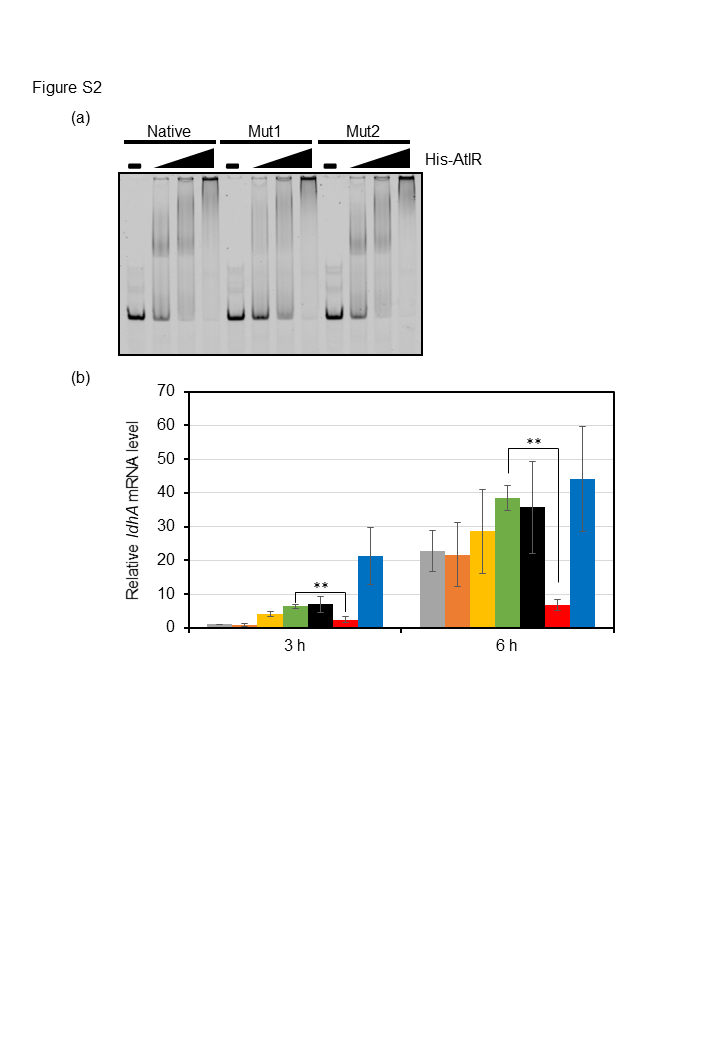

Supplement: Supplementary file 1 [file microorganisms-09-00550-s001.zip › Figure S2.TIF]
